# The Indirect Costs of the SARS-CoV-2 Pandemic: A Case of Severe Malaria in Brooklyn

**DOI:** 10.7759/cureus.12331

**Published:** 2020-12-27

**Authors:** Eitan Fleischman, Akil H Hutchinson, Nawar Z Paracha, Chathula Kumarasinghe, Eshan Patel

**Affiliations:** 1 Internal Medicine, NewYork-Presbyterian Brooklyn Methodist Hospital, Brooklyn, USA; 2 Hematology and Oncology, NewYork-Presbyterian Brooklyn Methodist Hospital, Brooklyn, USA

**Keywords:** falciparum malaria, severe malaria, sars-cov-2 and covid-19, indirect cost

## Abstract

Severe malaria due to the infection of *Plasmodium falciparum* is a critical infection that may lead to multisystem abnormalities if not promptly and adequately treated. We present a case of severe malaria in a patient recently repatriated from Conakry, Guinea, West Africa, marooned during the recent coronavirus disease 2019 (COVID-19) pandemic caused by severe acute respiratory syndrome coronavirus 2 (SARS-CoV-2). While the direct costs of the SARS-CoV-2 pandemic and its indirect effect on neighboring industries have been analyzed, the indirect costs of other ailments in medicine have yet to be fully established. This case explores the ramifications of the SARS-CoV-2 pandemic on what would otherwise have been routine prophylaxis of malaria in a traveler. Given the pandemic, the healthcare industry has had fundamental changes that have impacted access to healthcare, particularly in the outpatient setting.

## Introduction

Coronavirus disease 2019 (COVID-19) is caused by severe acute respiratory syndrome coronavirus 2 (SARS-CoV-2), an acute respiratory virus that has caused an unparalleled international health crisis. First noted in China in late December of 2019, the virus has now spread into an international pandemic [1]. Symptomatology ranges from asymptomatic to complex acute respiratory distress requiring mechanical ventilation and prolonged intensive care unit management. At time of publication, the World Health Organization (WHO) reports greater than 36.5 million confirmed cases and over 837 thousand associated deaths [2]. 

Due to rampant spread, high transmission rates, and acuity, COVID-19 has caused an unprecedented upset in numerous economic, educational, and medical sectors [3]. In healthcare, the medical provider’s focus has been shifted from routine follow-up, treatment, and health maintenance to COVID-19 prevention and treatment. From a patient perspective, fear of contraction, insurance loss, and reduced accessibility to medical care have inhibited patients from interfacing with the healthcare system. The lack of routine healthcare had led to a yet uncalculated indirect cost of the COVID-19 pandemic. We describe an unusual case of severe malaria in Brooklyn; a small microcosm of the indirect costs of the current COVID-19 pandemic.

## Case presentation

A 26-year-old male with no known past medical history presented with recurrent fevers, nausea, vomiting, myalgias, and decreased oral intake. He was recently repatriated from Conakry, Guinea after a three-month travel ban during the SARS-CoV-2 pandemic. Initial itinerary was for a two-week excursion and adequate malaria prophylaxis was obtained. Upon return, he delayed his presentation to the ED for five days due to fears of contracting SARS-CoV-2. On presentation the patient was found to be febrile to 39.4C. His blood smear was positive for *Plasmodium falciparum* with 14% infected red blood cells, thrombocytopenia of 25 K/uL, lactate dehydrogenase of 874 u/L and haptoglobin <31 mg/dL. He was started on a combination atovaquone and proguanil regimen and parasitology was monitored daily for decrease in infection rate.

Fevers and vomiting persisted throughout the beginning of his hospitalization. Repeat parasitology showed a decrease in infected cells to 7%. Thrombocytopenia improved (39 K/uL), however the patient became anemic (hemoglobin (Hgb) 15.1 g/dL to 11.4 g/dL) from admission. On day three there was concern for decreased bioavailability of treatment due to persistent emesis. Repeat parasitology showed a decrease of infected cells to 0.2%. The patient was continued on treatment for an additional day due to partial emesis on day two. On day four, parasitology showed 0.0% infected cells, and no blood parasites were observed. His symptoms resolved completely. The patient was subsequently discharged from the hospital without further complications and scheduled for outpatient follow-up.

## Discussion

In 1990 (with subsequent revisions in 2000), the WHO defined criteria for severe malaria as *Plasmodium falciparum*-infected erythrocytes percentage greater than 5%, severe anemia due to hemolysis, hemoglobinuria, cerebral malaria, acute kidney injury, acute respiratory distress syndrome (ARDS), pulmonary edema, jaundice, bleeding, hypotension, metabolic acidosis, and hypoglycemia [4]. The incubation time for *P. falciparum* malaria can vary between 12-14 days but may involve longer periods with inadequate prophylaxis. It is more often seen in industrialized countries as travelers from non-endemic countries returning from their travels [5]. 

When inadequate prophylaxis or resistance to prophylactic medications occurs, treatment is time-dependent and determines the severity of complications. Treatment of malaria depends on multiple factors which include the patient’s age and their degree of background immunity, the severity of infection, and susceptibility to antimalarial medications along with the cost and availability of these medications. In particular, the choice of treatment for *P. falciparum* depends on the sensitivity of the parasite to antimalarial medications in the place it was acquired. Infections acquired from North Africa, Middle East, Haiti, and Central America north of the Panama Canal are treated with chloroquine which is a three-day course of 25 mg/kg divided into three daily doses of 10 mg/kg for the first two days and 5 mg/kg on the third day. Amodiaquine is a more effective alternative in areas where there is a low resistance to chloroquine and is given as 35 mg/kg over three days [6]. Infections from chloroquine-resistant areas, like some regions of Asia and South America, are treated with a single-dose combination of pyrimethamine and a long-acting sulfonamide (most commonly sulfadoxine) [6]. Unfortunately, resistance to this combination has developed in Southeast Asia and South America [4]. The choice of treatment for multi-drug resistant *P. falciparum* is mefloquine, halofantrine, or quinine with tetracycline [6]. Artesunate, another restricted antimalarial which may be used for severe malaria treatment (2.4 mg/kg IV q12h x three doses, then 2.4 mg/kg for up to seven days) and distributed by the Centers for Disease Control and Prevention (CDC) in the US. Studies have shown that there is an increase in parasite clearance compared to quinine-based drugs [7]. The patient in this report was treated with Malarone for a total of four days but was evaluated for artesunate due to concerns of non-responsive parasitology despite treatment. Ultimately artesunate was not administered as red blood cell (RBC) involvement dropped from 7% to 0.2% following Malarone use. While treatment of malaria can be fairly uncomplicated, it is highly dependent on the availability of medications and acuity of care. The patient did not receive adequate prophylaxis and fear of SARS-CoV-2 led secondarily to an unanticipated cost of the current pandemic

The direct costs of the SARS-CoV-2 pandemic have been thoroughly studied, but indirect costs are far more insidious. The direct median costs for hospitalizations of acute COVID-19 infection in the US have been estimated to be $3,045 per person [3]. A proposed attack rate of 20% would account for greater than $163 billion over the acute infectious phase of the virus. Estimates of this magnitude assess the direct acute costs of COVID-19 and do not account for rehabilitation and recovery. The indirect costs of untreated comorbidities, undiagnosed illness, and lack of preventive care for the rest of the healthcare sector have yet to be fully studied. 

Indirect costs are the measure of expense not directly attributable to the original cost. It is often an unforeseen trickle-down pertaining to the inciting event that does not become readily apparent until later. It may be calculated as not only a financial burden, but the emotional and social costs to the patient and caregivers as well. The indirect costs of COVID-19 are being studied in sectors such as education, finance, and agriculture: however, little is yet known about the indirect healthcare costs associated with the COVID-19 pandemic [8,9]. Amimo et al. propose a potential increase and severity of endemic HIV, malaria, and tuberculosis in Africa secondary to COVID-19 and lack of treatment on current endemic ailments [10]. Chronic medical conditions such as congestive heart failure and obesity, already large burdens on the US healthcare system, may not have been effectively treated, and the lapse of care during this time would only propel these comorbid costs [3,11].

## Conclusions

The lack of proper personal protective equipment, fear of contraction, reduced transportation, and abbreviated outpatient medicine has led to a yet unmeasured decrease in management of acute and chronic disease in both the outpatient and inpatient settings. Patients such as the one described in this article have had their access to routine healthcare abruptly stopped due to COVID-19. It is therefore incumbent to consider not only the direct costs of the recent pandemic but attempt to anticipate the indirect ramifications as well. 
